# Adjuvant chemotherapy for lymph node positive esophageal squamous cell cancer: The prediction role of low mean platelet volume

**DOI:** 10.3389/fonc.2022.1067682

**Published:** 2022-12-06

**Authors:** Xiaoling Liu, Kaijiong Zhang, Jie Tang, Li Jiang, Yu Jiang, Qifeng Wang

**Affiliations:** ^1^ Departments of Medical Oncology, Cancer Center, West China Hospital, Sichuan University, Chengdu, Sichuan, China; ^2^ Department of Medical Oncology, Sichuan Cancer Hospital and Institute, Sichuan Cancer Center, School of Medicine, University of Electronic Science and Technology of China, Chengdu, China; ^3^ Department of Laboratory Medicine, Sichuan Cancer Hospital and Institute, Sichuan Cancer Center, School of Medicine, University of Electronic Science and Technology of China, Chengdu, China; ^4^ Department of Radiation Oncology, Sichuan Cancer Hospital and Institute, Sichuan Cancer Center, School of Medicine, University of Electronic Science and Technology of China, Chengdu, China

**Keywords:** esophageal squamous cell cancer, lymph node positive, mean platelet volume, adjuvant chemotherapy, prognosis

## Abstract

**Background:**

This study aimed to examine whether MPV is a useful prognostic marker and investigated whether MPV is a risk factor that helps identify patients with locally advanced-stage ESCC who will most likely benefit from adjuvant chemotherapy.

**Methods:**

Patients (n =1690) with histologically confirmed ESCC were diagnosed with locally advanced stage (pT3-4N0M0 and pT1-4N+M0) at Sichuan Cancer Hospital from 2009 to 2017. Clinicopathological factors and platelet-related values were tested for their associations with survival using univariate and multivariate Cox regression analyses. The optimal cut-off value for continuous variables was determined using the ‘maxstat’ R package. The KM curve continuous variable analysis was performed to identify the optimal cut-off value for MPV. Cumulative survival rates were determined using the Kaplan–Meier estimator and compared using the log-rank test. The survival analysis was performed using the ‘survival’ R package. All statistical analyses were performed using R software 4.1.3 (https://www.r-project.org/), and a two-sided p-value <0.05 was considered to indicate statistical significance.

**Results:**

Multivariate analysis indicated that low MPV was an important risk factor for overall survival in locally advanced ESCC, independent of classic clinicopathological factors. The optimal cut-off value of MPV (11.8 fL) was used to stratify high-risk patients. Patients with low mean platelet volumes had a worse prognosis than those with larger platelet volumes, according to Kaplan–Meier analysis and the log-rank test. Patients diagnosed with a pathological lymph node-positive stage with a low MPV (≤11.8 fL) benefited from postoperative chemotherapy, but not those with a high-level MPV (>11.8 fL).

**Conclusion:**

MPV served as an independent predictor of prognosis of locally advanced-stage ESCC and predicted a survival benefit conferred by postoperative adjuvant chemotherapy in lymph node-positive ESCC.

## Introduction

Esophageal cancer is one of the most lethal malignancies that accounted for 604000 new cases and 544000 deaths in 2018 worldwide (1). In China, esophageal squamous cell carcinoma (ESCC) is the predominant histological type, and its incidence and mortality rate rank sixth and fourth, respectively (2). Surgical resection is the mainstay treatment of non-distant metastatic ESCC. Although neoadjuvant chemoradiotherapy is recommended in locally advanced (T3–4N0M0 or lymph node-positive) ESCC to improve survival (3), many patients still undergo surgery as their initial treatment (4). The overall prognosis is bleak. Surgery alone is unsatisfactory for patients who have not receive preoperative therapy. The five-year survival rates were only 15 percent for patients with lymph node positive patients. Therefore, postoperative chemotherapy is recommended to control distant micro metastatic disease (5). However, uncontrolled trials and retrospective comparisons reported different outcomes, not all locally advanced ESCC who have not receiving neoadjuvant therapy patients derive a survival benefit from adjuvant chemotherapy, even with lymph node stratification (6–8). Because of the inconsistent results, the issue of postoperative adjuvant therapy still not be addressed. Suggesting that a reliable and widely available predictive marker is required beyond the current staging system.

The mean platelet volume (MPV) is a precise measurement of platelet size, which reflects changes in the levels of platelet stimulation or rates of platelet production (9). Altered MPV levels, which are found in many solid malignancies, provide important prognostic information for cancer patients (10–12). Studies on the relationship between MPV levels and prognosis of ESCC are rare and report divergent results (13, 14). Besides, most of the studies on MPV and tumor describe its relationship with poor prognosis. Little has been done to explore the possibility that MPV may influence the decision making of treatment. Therefore, the aim of this study was to evaluate whether the MPV serves as a useful prognostic marker as well as a risk factor to help identify patients with locally advanced-stage ESCC who will most likely benefit from adjuvant chemotherapy.

## Patients and methods

### Patients

Patients (n = 3210) with esophageal cancer underwent esophagectomy at Sichuan Cancer Hospital (Chengdu, China) from January 2009 to December 2017. The inclusion criteria were as follows: (1) Post-histologically confirmed ESCC with non-distant metastasis patients without previous anticancer therapy, (2) non-cervical esophageal cancer, (3) complete tumor resection (R0), (4) optional adjuvant chemotherapy, (5) pathological lymph node-positive (pT1-4N+M0); (6) pathological T3 or T4N0M0 staging (pT3-4N0M0), and (7) complete clinical and follow-up data. The exclusion criteria were as follows: (1) Patients with pathological T1-2N0M0 stage, (2) other malignancies or perioperative mortality, and (3) Patients received post-operative adjuvant radiotherapy, (4) follow-up <3 months. We retrospectively analyzed patients (n = 1690) with locally advanced-stage (pT3-4N0M0 and pT1-4N+M0) ESCC. The final clinical follow-up examination was completed on December 31, 2019, and the median follow-up was 60 months. The Institutional Ethics Committee of Sichuan Cancer Hospital approved this study.

### Diagnosis and treatment

After acquiring a detailed history and performing a complete physical examination, venous blood was taken from patients for routine hematological analyses. The conventional staging procedures were based on neck, chest, and abdomen computed tomography (CT) with contrast, radio nucleotide bone scan, brain magnetic resonance imaging, endoscopic ultrasound and barium swallow. Positron emission tomography (PET)/CT was optional for suspicious distant metastases. McKeown or Ivor-Lewis esophagectomy was administered to patients with no distant metastases confirmed by imaging. Postoperative adjuvant chemotherapy was selected for patients with traditional high-risk pathological factors (e.g., lymph node involvement, T3-4 advanced stage, vascular or nerve invasion, or poor histological differentiation). Surgery alone (Surgery) was performed on 948 patients, and 742 patients received adjuvant chemotherapy (S+CT), typically initiated 4–6 weeks after surgery. Chemotherapy regimens were mainly dual drugs containing Carboplatin or Cisplatin, or fluorouracil alone, depending on a patient’s physical condition.

### Analysis of blood samples and data collection

We used an automated blood cell counter to measure complete blood counts and platelet-related values of EDTA-treated blood specimens (Sysmex XE-2100, Japan). Data of patients were acquired for sex, age, and Karnofsky Performance Status (KPS) score. Platelet-related variables in preoperative routine hematological tests included as follows: platelet count(PLT), platelet distribution width(PDW), MPV, and platelet hematocrit(PCT). Pathological characteristics such as tumor location, tumor length, tumor grade, vascular or nerve invasion, T stage, N stage, and survival time were obtained from patients’ postoperative medical records. Overall survival (OS) was defined as the length of time from surgery to censoring or death.

### Statistical analysis

The significance of differences between categorical variables were compared using the chi-squares test or Fisher’s exact test when appropriate. Cox proportional hazard models were used to calculate hazard ratios (HRs) with 95% confidence intervals (CIs) and estimate the association between clinicopathological, platelet-related factors with OS. Univariate analysis was carried out using Cox regression analysis with the clinicopathological and platelet-related characteristics. Variables with a P value <0.1 in the univariate analysis results was used in multivariate analysis. Kaplan–Meier method was used to analyze cumulative survival rates and generate survival curves. All statistical analyses were performed using R software 4.1.3 (https://www.r-project.org/), and a two-sided p-value <0.05 was considered to indicate statistical significance. OS was the primary study endpoint.

## Results

### Patients’ clinicopathological characteristics

The clinicopathological characteristics of patients are summarized in **Table 1**. Patients with ESCC (n = 1690) receiving esophagectomy met the inclusion criteria and were diagnosed with locally advanced-stage disease. The median age was 62 years, and their median survival was 38.7 (95% CI 36–42.6) months (**sFigure 1**). 65.1% (1101/1690) had lymph node metastasis (pT1-4N+M0), 34.9% (589/1690) were diagnosed with pT3-4N0M0 stage. Male patients accounted for 83% (1403/1690) of the group, and 53.4% (902/1690) of patients had mid-thoracic esophageal cancer. Tumors longer than 4 cm were present in 57.6% (973/1690) of patients. The percentages of patients with T1-2 and T3-4 were 15.6% (263/1690) and 84.4% (1427/1690), respectively, and 19.5% (330/1690) and 22.2% (375/1690) were pathologically diagnosed with vascular or nerve invasion after surgery, respectively. 43.9% (742/1690) of patients received adjuvant chemotherapy after surgery.

**Table 1:** Clinico-pathological characteristics of local advanced stage ESCC by MPV levels.

Characteristics	All patients *N=1690*	MPV ≤ 11.8fL *N=1005*	MPV>11.8fL *N=685*		p. overall
age					0.625
<62	793 (46.9%)	477 (47.5%)		316 (46.1%)	
≥62	897 (53.1%)	528 (52.5%)		369 (53.9%)	
Sex					<0.001***
male	1403 (83.0%)	867 (86.3%)		536 (78.2%)	
female	287 (17.0%)	138 (13.7%)		149 (21.8%)	
KPS score					0.682
70-80	754 (44.6%)	453 (45.1%)		301 (43.9%)	
90-100	936 (55.4%)	552 (54.9%)		384 (56.1%)	
Tumor length					0.013 **
<4cm	717 (42.4%)	401 (39.9%)		316 (46.1%)	
>=4cm	973 (57.6%)	604 (60.1%)		369 (53.9%)	
Tumor Grade					0.795
Moderate	714 (42.2%)	419 (41.7%)		295 (43.1%)	
Poor	674 (39.9%)	402 (40.0%)		272 (39.7%)	
Well	302 (17.9%)	184 (18.3%)		118 (17.2%)	
Tumor location					0.551
Upper	393 (23.3%)	229 (22.8%)		164 (23.9%)	
Middle	902 (53.4%)	532 (52.9%)		370 (54.0%)	
Lower	395 (23.4%)	244 (24.3%)		151 (22.0%)	
Vascular invasion					0.595
no	1360 (80.5%)	804 (80.0%)		556 (81.2%)	
yes	330 (19.5%)	201 (20.0%)		129 (18.8%)	
Nerve invasion					0.953
no	1315 (77.8%)	781 (77.7%)		534 (78.0%)	
yes	375 (22.2%)	224 (22.3%)		151 (22.0%)	
T stage					0.371
T1	55 (3.3%)	33 (3.28%)		22 (3.21%)	
T2	208 (12.3%)	121 (12.0%)		87 (12.7%)	
T3	1279 (75.7%)	753 (74.9%)		526 (76.8%)	
T4	148 (8.7%)	98 (9.75%)		50 (7.30%)	
N stage					0.263
N0	589 (34.9%)	339 (33.7%)		250 (36.5%)	
N+	1101 (65.1%)	666 (66.3%)		435 (63.5%)	
Dis LN number					0.228
<10	136 (8.1%)	88 (8.8%)		48 (7.00%)	
>=10	1554 (91.9%)	917 (91.2%)		637 (93.0%)	
PLT					<0.001 ***
<177	844 (49.9%)	309 (30.7%)		535 (78.1%)	
>=177	846 (50.1%)	696 (69.3%)		150 (21.9%)	
PDW					<0.001 ***
<16.4	817 (48.3%)	608 (60.5%)		209 (30.5%)	
>=16.4	873 (51.7%)	397 (39.5%)		476 (69.5%)	
PCT					<0.001***
<0.2	746 (44.1%)	365 (36.3%)		381 (55.6%)	
>=0.2	944 (55.9%)	640 (63.7%)		304 (44.4%)	
treatment					0.566
Surgery	948 (56.1%)	570 (56.7%)		378 (55.2%)	
S+CT	742 (43.9%)	435 (43.3%)		307 (44.8%)	
TNM class					0.263
T3-4N0M0	589 (34.9%)	339 (33.7%)		250 (36.5%)	
T1-4N+M0	1101 (65.1%)	666 (66.3%)		435 (63.5%)	

MPV, Mean Platelet Volume; PLT, Platelet Count; PDW, Platelet Distribution Width; PCT, Platelet hematocrit; T, Tumor; N, lymph node; N+, Patients with pathology lymph node metastasis; KPS, Karnofsky performance scale; S+CT, Surgery followed by chemotherapy. **P< 0.01, ***P< 0.001.

### Univariate and multivariate analyses for OS

**Figure 1:**
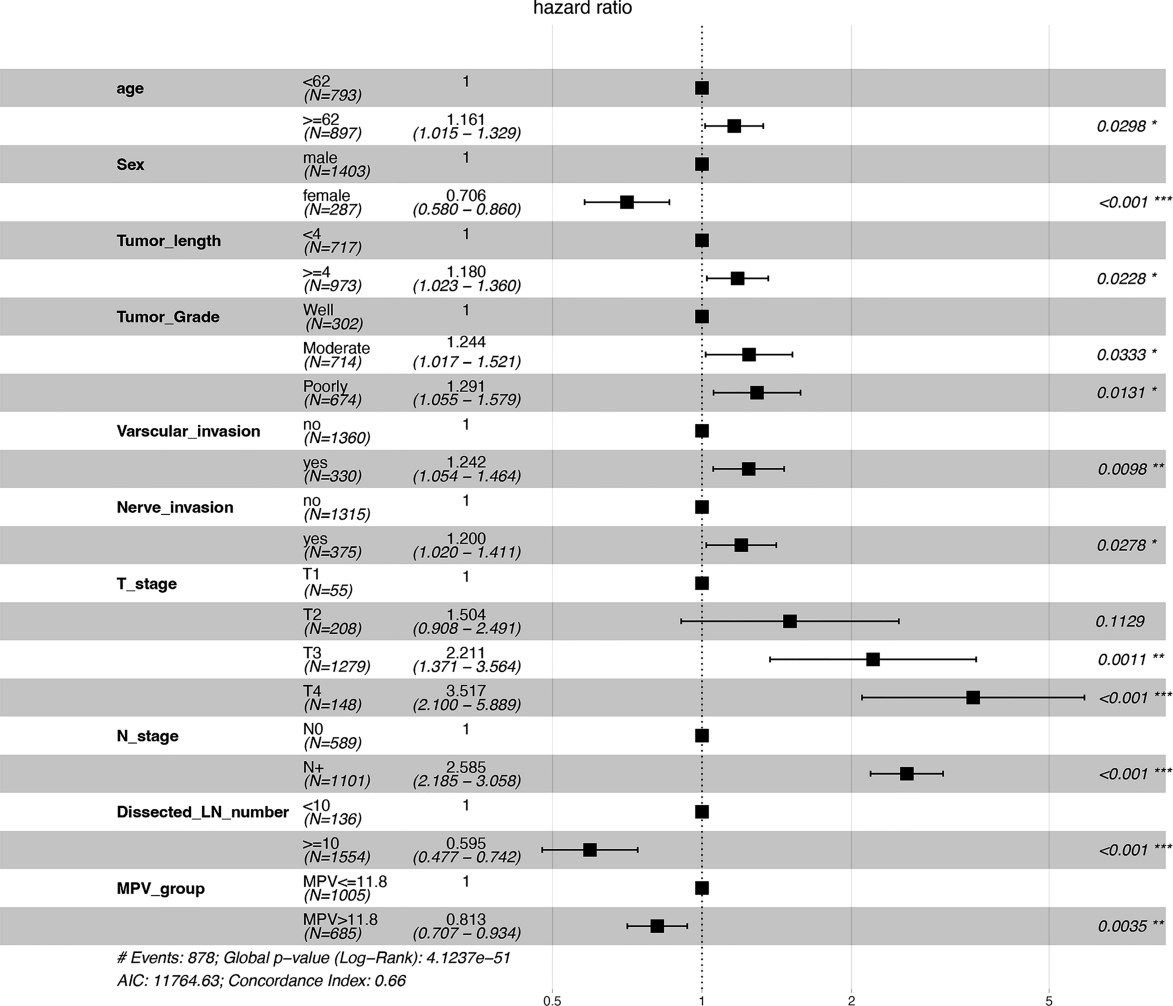
Forest plot of predictive factors associated with overall survival from the multivariate analysis. *P < 0.05, **P < 0.01, ***P < 0.001.

Univariate and multivariate analyses were carried out using Cox proportional hazards model with the following variables: age, sex, KPS, tumor length and grade, tumor location, T and N stage, nerve and vascular invasion, dissected lymph node numbers, MPV group, PCT, PDW and PLT (**Table 2**). A multivariate analysis was performed with the statistically significant parameters from the univariate analysis. These analyses demonstrated that age, sex, tumor length, tumor grade, vascular invasion, nerve invasion, T stage, N stage, dissected lymph node numbers, and MPV were independent prognosis predictors for OS (**Figure 1**). While the KPS score, tumor location, PCT, PDW, PLT were not associated with survival. The association between MPV and survival of ESCC therefore requires further investigation.

**Table 2:** Univariate and multivariate analyses of predictive factors associated with overall survival.

Characteristics		Univariate analysis			Multivariate analysis	
		HR.CI95.		p.value.x	HR.CI95.y	p.value.y
**1**	Age		1.17 (1.024-1.337)	0.021*	1.165 (1.018-1.333)	0.0265*
**2**	Sex		0.662 (0.545-0.804)	<0.001***	0.706 (0.58-0.86)	<0.001***
**3**	KPS score		1.003 (0.877-1.146)	0.972		
**4**	Tumor length		1.24 (1.08-1.424)	0.002**	1.174 (1.019-1.353)	0.0262*
**5**	Tumor Grade		1.184 (1.082-1.297)	<0.001***	1.114 (1.015-1.223)	0.0236*
**6**	Tumor location		0.958 (0.871-1.054)	0.375		
**7**	Vascular invasion		1.645 (1.407-1.922)	<0.001***	1.247 (1.058-1.469)	0.0084**
**8**	Nerve invasion		1.437 (1.232-1.675)	<0.001***	1.214 (1.034-1.427)	0.0181*
**9**	T stage		1.387 (1.217-1.581)	<0.001***	1.519 (1.337-1.726)	<0.001***
**10**	N stage		2.403 (2.053-2.813)	<0.001***	2.609 (2.21-3.079)	<0.001***
**11**	Dissected LN number		0.723 (0.583-0.896)	0.003**	0.594 (0.476-0.741)	<0.001***
**12**	MPV group		0.777 (0.677-0.892)	<0.001***	0.815 (0.709-0.936)	0.0039**
**13**	PCT		1.033 (0.904-1.18)	0.632		
**14**	PDW		0.94 (0.823-1.074)	0.366		
**15**	PLT		1.056 (0.925-1.205)	0.424		

*P< 0.05, **P< 0.01, ***P< 0.001.

### Optimal MPV cut-off value

**Figure 2:**
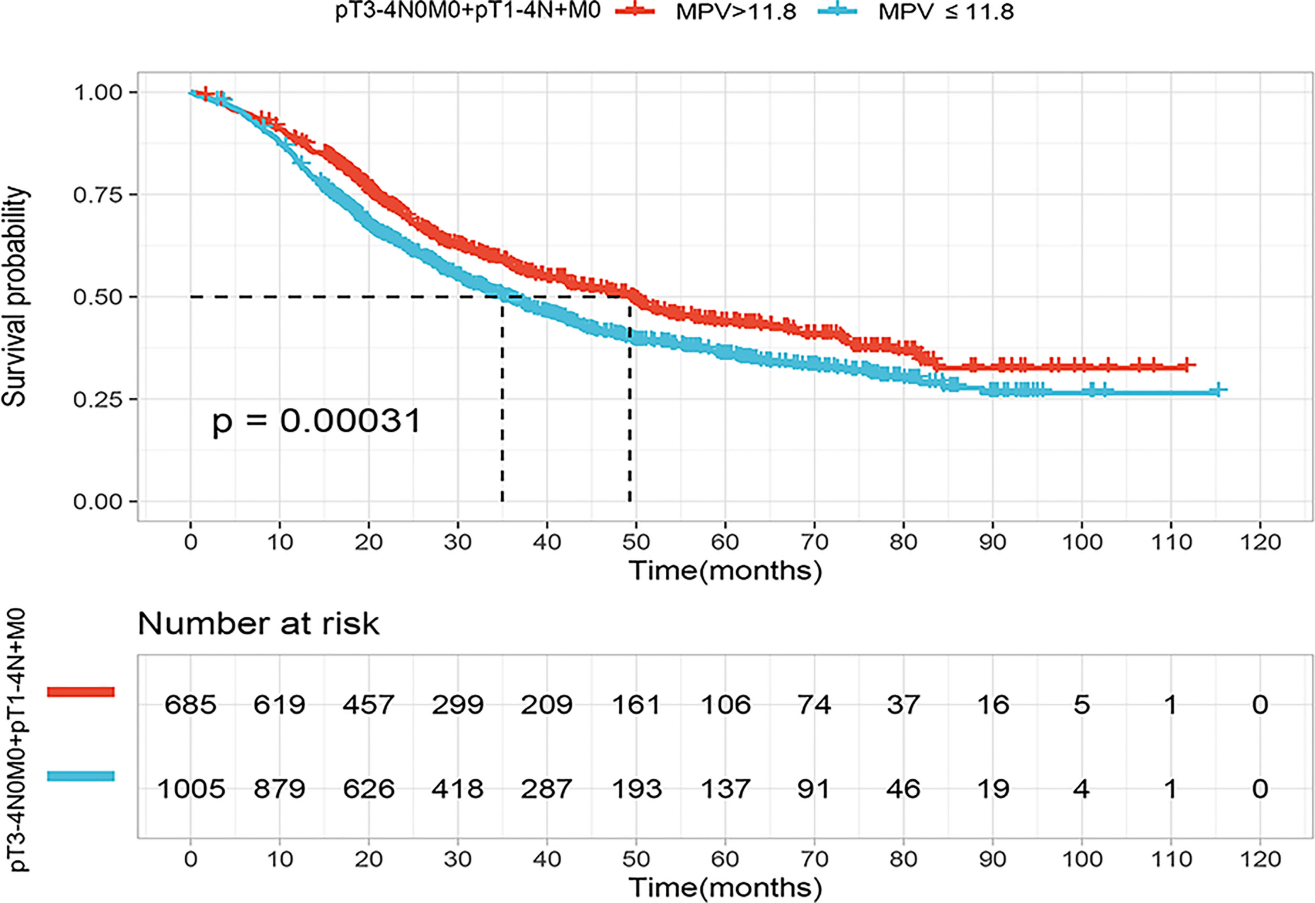
Kaplan–Meier curves for overall survival (OS) of the patients according to the mean platelet volume (MPV) cutoff value (11.8fL) in the locally advanced cohort (pT3-4N0M0+pT1-4N+M0).

According to KM curve continuous variable analysis, the optimal cut-off value for MPV was 11.8 fL (**sFigure 2**). The correlation between MPV levels (≤11.8 fL and >11.8 fL) and clinicopathological parameters are displayed in **Table 1**. The MPV level was significantly associated with sex (p<0.001) and tumor length (p=0.013). Survival analysis revealed patients with locally advanced ESCC with MPV >11.8 fL had a better prognosis than those with MPV ≤11.8 fL, the median survival was 49.3 months (95% CI, 42.1–56.5) in the MPV >11.8 fL group and 35 months (95% CI, 31.3–38.9) in the MPV ≤11.8 fL group, respectively (p<0.001) (**Figure 2**).

### Survival comparisons according to MPV

The survival of patients diagnosed with locally advanced ESCC who received adjuvant chemotherapy (n = 742) or not (n = 948) was analyzed using the Kaplan–Meier method (**sFigure 3A**). We found that adjuvant chemotherapy increased the OS of patients in both diagnosed with stage pT3-4N0M0 or pT1-4N+M0. Median survival of locally advanced stage was 46.3 months (95% CI, 39.9–55.6) in the surgery followed by chemotherapy group and 35.5 months (95% CI, 31.5–39.3) in surgery alone group, respectively (p<0.001). Patients with pT3-4N0M0 stage had a median survival of 65.4 months (95% CI, 49.4–84.4) in receiving surgery alone group and median survival exceeded the current follow up time (**sFigure 3B**). Patients with pT1-4N+M0 stage receiving adjuvant chemotherapy or surgery alone had a survival of 31.1 months (95% CI, 27.9–36.1) and 27.6 months (95% CI, 24.1–32.7) respectively (**sFigure 3C**).

**Figure 3:**
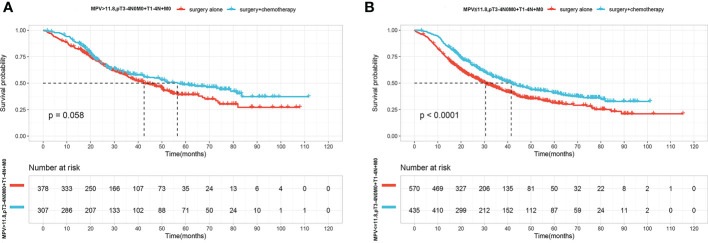
**(A)** OS curves for patients with Surgery(S) and Surgery followed by chemotherapy(S+CT) of MPV value over 11.8fL. **(B)** OS curves for patients with S and S+CT of MPV value over less than 11.8fL.

Since patients with lower MPV values had a poor prognosis. We therefore asked if low MPV serves as an important predictor to guide postoperative adjuvant chemotherapy in locally advanced ESCC. In the high MPV group (MPV >11.8 fL), OS did not significantly differ according to adjuvant chemotherapy(P=0.058) (**Figure 3A**). In the low MPV group (MPV ≤11.8 fL), OS was 41.5 months (95% CI, 36.1–49.6) of patients receiving adjuvant chemotherapy and 30.5 months (95% CI, 27.3–35.1) for those who underwent surgery alone. Patients with low MPV values who received adjuvant chemotherapy lived significantly longer than those who underwent surgery alone (**Figure 3B**).

**Figure 4:**
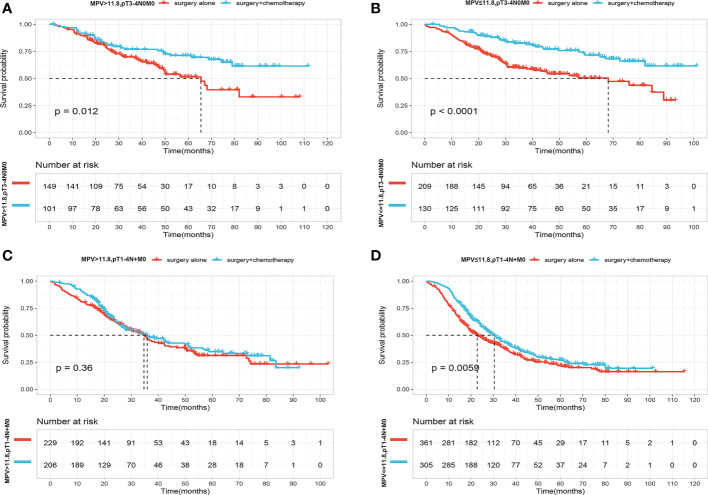
**(A)** OS curves for patients with p T3-4N0M0 stage with S and S+CT of MPV value over 11.8f. **(B)** OS curves for patients with p T3-4N0M0 stage with S and S+CT of MPV value less than 11.8fL. **(C)** OS curves for patients with pT1-4N+M0 stage with S and S+CT of MPV value over 11.8fL. **(D)** OS curves for patients with p T1-4N+M0 stage with S and S+CT of MPV value less than 11.8fL.

We next asked who benefited the most from adjuvant chemotherapy according to MPV values. For this purpose, we conducted more detailed Kaplan–Meier analysis according to pathological stage pT3-4N0M0 or pT1-4N+M0. Adjuvant chemotherapy provided a survival benefit for patients with pT3-4N0M0 regardless of the MPV value (**Figures 4A, B**). The OS curves did not differ after stratification according to MPV >11.8 fL, and adjuvant chemotherapy did not improve outcomes for patients with pT1-4N+M0 stage (**Figure 4C**). However, for patients with pT1-4N+M0 stage, considering preoperative MPV <11.8 fL, median survival was 22.8 months (95% CI, 19.4–27.9) in the surgery-alone group and 30.4 months (95% CI, 26.5–34.9) in the surgery-combined adjuvant chemotherapy group. Adjuvant chemotherapy prolonged median survival in the low MPV group by 7.6 months (P=0.006) (**Figure 4D**).

## Discussion

In the present study, we analyzed the survival of patients with locally advanced pathological stages T3-4N0M0 and T1-4N+M0. This subset of patients had variable prognoses. The results of salvage chemotherapy were controversial (6–8). Previous results on the efficacy of adjuvant chemotherapy conflict or include a limited number of cases (15–17). Thus, whether adding chemotherapy to surgery for ESCC remains under investigation. Moreover, this problem may be explained by the absence of a reliable marker to predict patient population that will experience an absolute benefit conferred by adjuvant chemotherapy.

Several studies identified high-risk clinicopathological factors for esophageal cancer (18, 19). Here we show that sex and age, T and N stage, nerve and vascular invasion, tumor length and grade, dissected lymph node numbers, as well as MPV, confer significant prognostic value. Moreover, the MPV had highly statistically significant coefficient compared with traditional pathological risk factors such as tumor grade, tumor location, tumor length, and nerve and vascular invasion. As a routinely available and inexpensive means of hematological analysis, the significance of the MPV in esophageal cancer deserves further investigation.

We identified here the optimum cut-off value (11.8 fL) of the MPV to stratify increased risks. Patients with low MPV values (≤11.8 fL) experienced a 14.3-month reduction in OS compared with patients with high MPV values (>11.8 fL). Consistent among most studies, a low MPV is a negative factor for survival of patients with solid tumors (11–13). However, the results of a study with a background similar to that of the present study show a completely different meaning of the MPV (14). To the best of our knowledge, the present study analyzed the largest sample size to investigate the prognostic and predictive values of the MPV. Specifically, we found that the reduction but not elevation of MPV was significantly associated with poor survival in locally advanced ESCC.

The underlying mechanisms of low MPV values associated with poor prognosis in cancer is unknown. Related to the physiology of platelet production, MPV is inversely associated with the platelet count, and differences in the MPV compensate for variations in platelet numbers and describe a constant platelet mass (20). Multiple studies show that elevated platelet counts occur in advanced cancers (21, 22). This may be explained by elevated platelet numbers that are triggered by the release of specific growth factors from a tumor. Furthermore, ongoing inflammation associated with cancer development and exacerbation is well documented (23). In contrast, platelets generated in this way limit the activity of natural killer cells and shield tumor cells from recognition by the immune system (24, 25). Moreover, tumor-educated platelets release platelet-derived growth factor, vascular endothelial growth factor, and transforming growth factor–β1 to suppress the antitumor immune response (26, 27). In particular, we do not know whether decreased platelet size is the cause or consequence of tumor progression. Pretreatment small MPV was associated with worse clinicopathologic features (larger tumor) in renal cell carcinoma (28). Our analysis found that low MPV was related with a longer tumor length. A longer tumor length in esophageal cancer has a worse prognosis. Sufficient tumors maybe a prerequisite for affecting the platelet volume. These interesting but unclear relationships are really worthy of our further exploration.

There are other possible explanations. For example, platelet surface molecules bind and transport tumor-derived secreted membrane vesicles to promote metastasis (29). Tumor-educated smaller platelets exhibit stronger prothrombotic capacity, leading to an increased risk of venous thromboembolism and death (30). Increased platelet wear or shear forces in advanced stages of disease may lead to a decrease in the MPV. There are few studies on the mechanism of MPV changes in ESCC. The altered transcriptome of the bone marrow megakaryocyte lineage or changes in cytokines levels in serum are worthy of attention. Thus, there is an urgent requirement for laboratory studies on the mechanism underlying the change in platelet size in locally advanced ESCC.

Chemotherapy kills not only cancer cells but also contributes to an immunosuppressive environment (31, 32), representing a double-edged sword. Thus, the risk-to-benefit ratio should be considered. Detailed knowledge and screen of patients may reduce or avoid damage caused by adjuvant chemotherapy. Furthermore, the outcomes of adjuvant chemotherapy in ESCC are conflicting. Most reports show a benefit for disease-free survival but not OS (6, 8, 15, 17). Here we analyzed the survival of patients with ESCC with locally advanced-stage disease, specifically pT3-4N0M0 and pT1-4N+M0. Kaplan–Meier analysis of OS shows that these patients benefited from adjuvant chemotherapy. Considering the conflicting results of previous clinical trials, it is difficult to determine the patients who will benefit from adjuvant therapy and how to mitigate its adverse effects. Here we found that a combined low MPV (≤11.8 fL) of patients diagnosed with a lymph node-positive stage significantly benefited from postoperative adjuvant chemotherapy. Conversely, adjuvant chemotherapy did not confer a survival benefit upon patients with a lymph node-positive stage with high MPV values.

The median survival of patients with ESCC with an earlier stage (pT3-4N0M0) is >5 years. Adjuvant chemotherapy prolongs the survival of such patients regardless of their MPV values, and to make matters more complex, the prognostic and predictive significance of MPV appears suboptimal. Here we show that 547 of 589 of patients were actually pT3N0M0 stage (AJCC 8^th^ IIA stage). This may possibly be explained by the very weak ability of early-stage tumors to affect platelets. Platelets in their early stage have not been educated by tumors, and platelet size cannot reflect tumor progression or the response to antitumor therapy. To our knowledge, there are no studies comparing the differences in platelet function between patients with early or advanced ESCC. Our present findings therefore suggest that platelet functions of these patients may differ according to disease stage.

The present study has certain limitations. First, selection bias is indeed an inherent weakness of retrospective studies. Our analysis was based on data obtained from a single institution, the results may be affected by unit-specific practices. Using this MPV cut-off value for clinical decision making requires data support and validation from more centers. Second, the changes of MPV values are likely to provide us with more prognostic and predictive information, and we did not follow up the MPV after surgery and chemotherapy. Therefore, △MPV of patients before and after treatment might be a good indicator for prognosis and prediction of esophageal cancer. Furthermore, most analyzed cases included patients treated before 2017, when the prevailing treatment model prioritized surgery, which differs from the current treatment mode of concurrent radiotherapy and chemotherapy followed by surgery. However, >50% of patients undergo surgery as their initial treatment in the real world. Therefore, our work promises to significantly assist clinical decision-making.

## Conclusion

Our results show that low MPV served as a negative prognostic factor in locally advanced-stage ESCC. Moreover, as a high-risk factor, low MPV may contribute to rigorous screening for lymph node-positive staging of patients with ESCC who receive adjuvant chemotherapy.

## Data availability statement

The original contributions presented in the study are included in the article/**Supplementary Material**. Further inquiries can be directed to the corresponding author.

## Ethics statement

The studies involving human participants were reviewed and approved by the institutional ethics committee of Sichuan Cancer Hospital. The ethics committee waived the requirement of written informed consent for participation.

## Author contributions

Conceptualization and design, QW and XL. Supervision support, QW. Statistical analyses and original drafting of the manuscript, XL and KZ. Methodology, review and editing of the paper, JT, YJ and LJ. Data curation and editing of the paper, XL. Final approval of manuscript for submission, all authors. All authors contributed to the article and approved the submitted version.
