# Supplementary figures and images for: Adjuvant chemotherapy for lymph node positive esophageal squamous cell cancer: The prediction role of low mean platelet volume
Source: Front Oncol. 2022 Dec 6;12:1067682. doi: 10.3389/fonc.2022.1067682 (PMC9763308; doi:10.3389/fonc.2022.1067682)

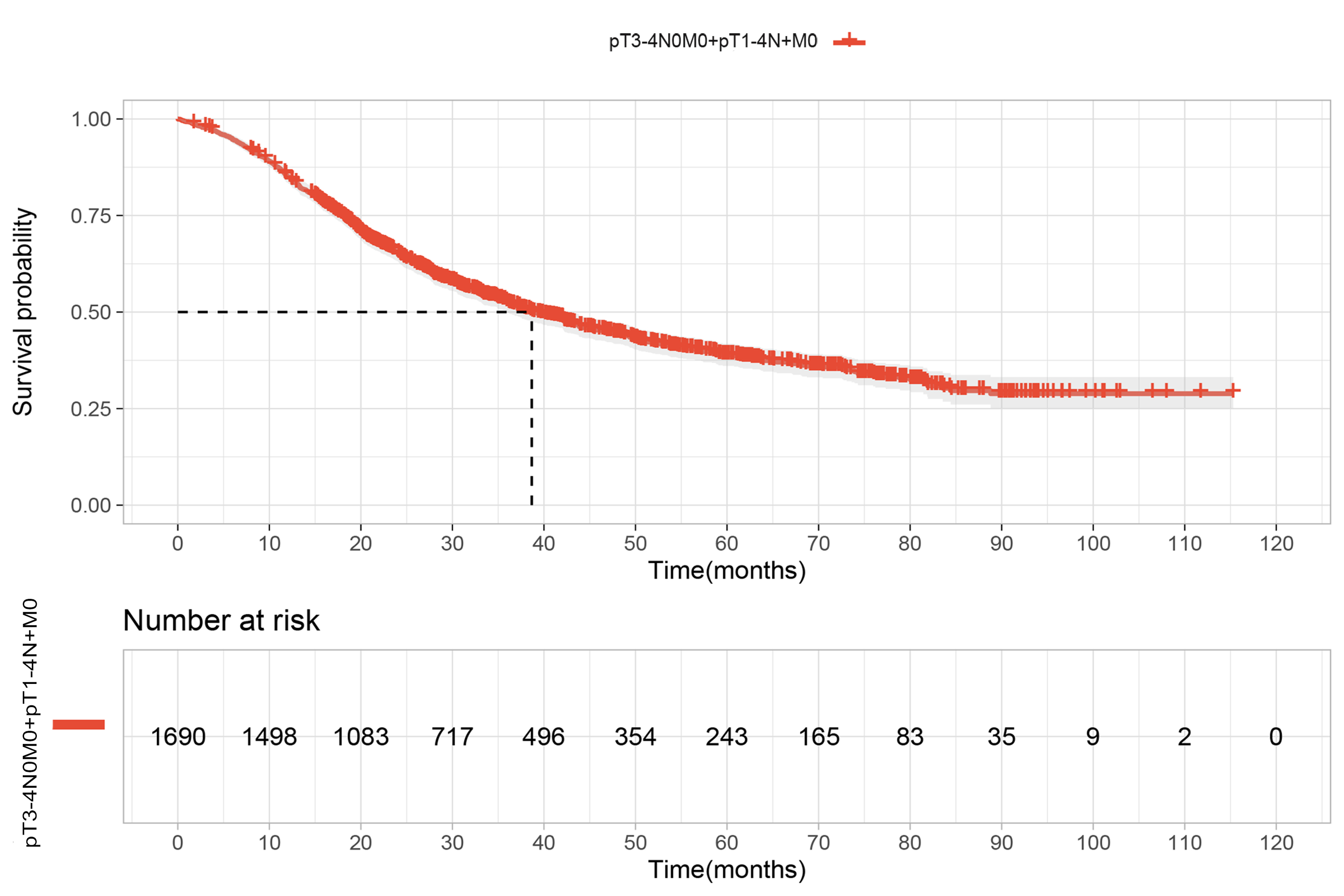

Supplement: Supplementary file 1 [file Image_1.jpeg]

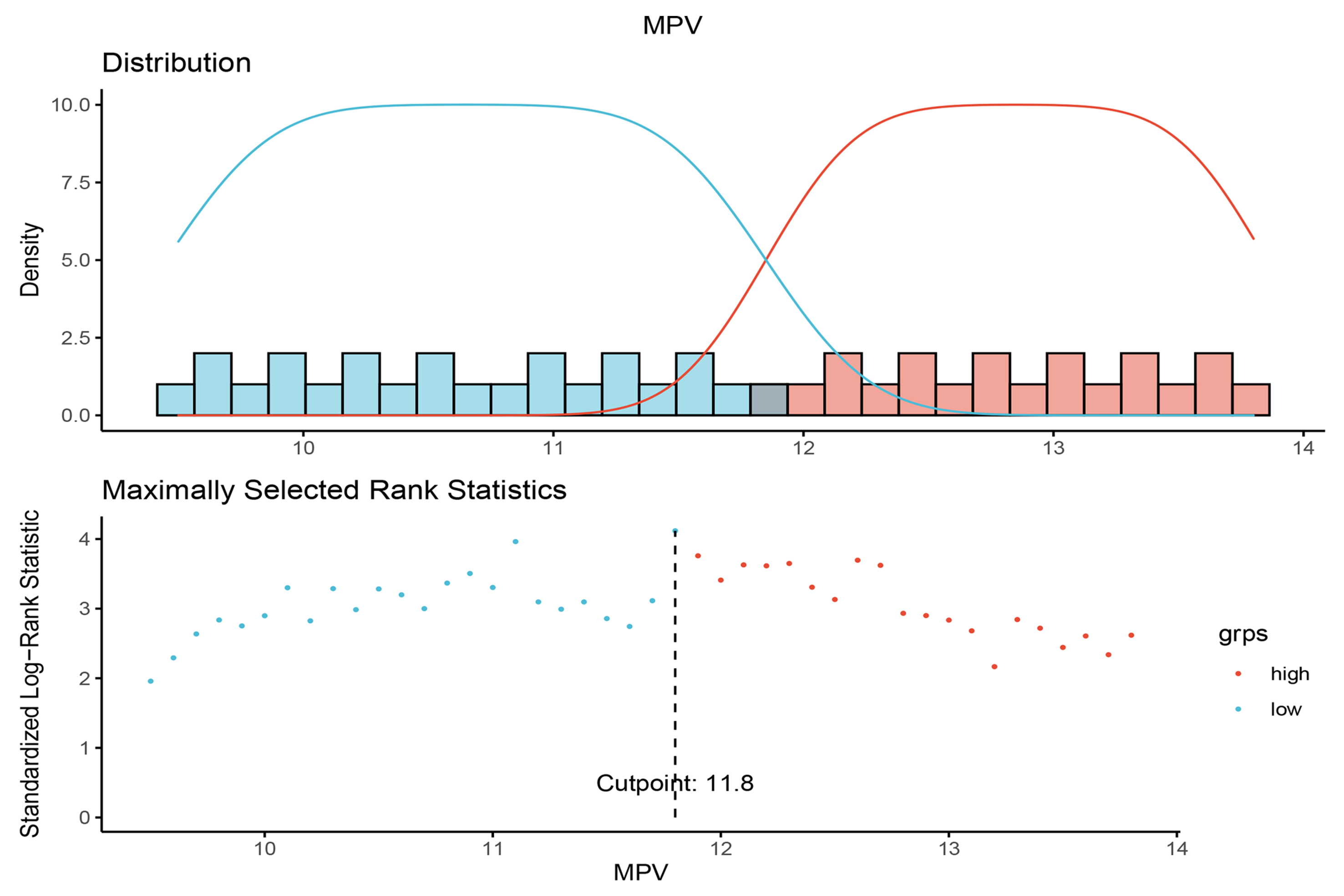

Supplement: Supplementary file 2 [file Image_2.jpeg]

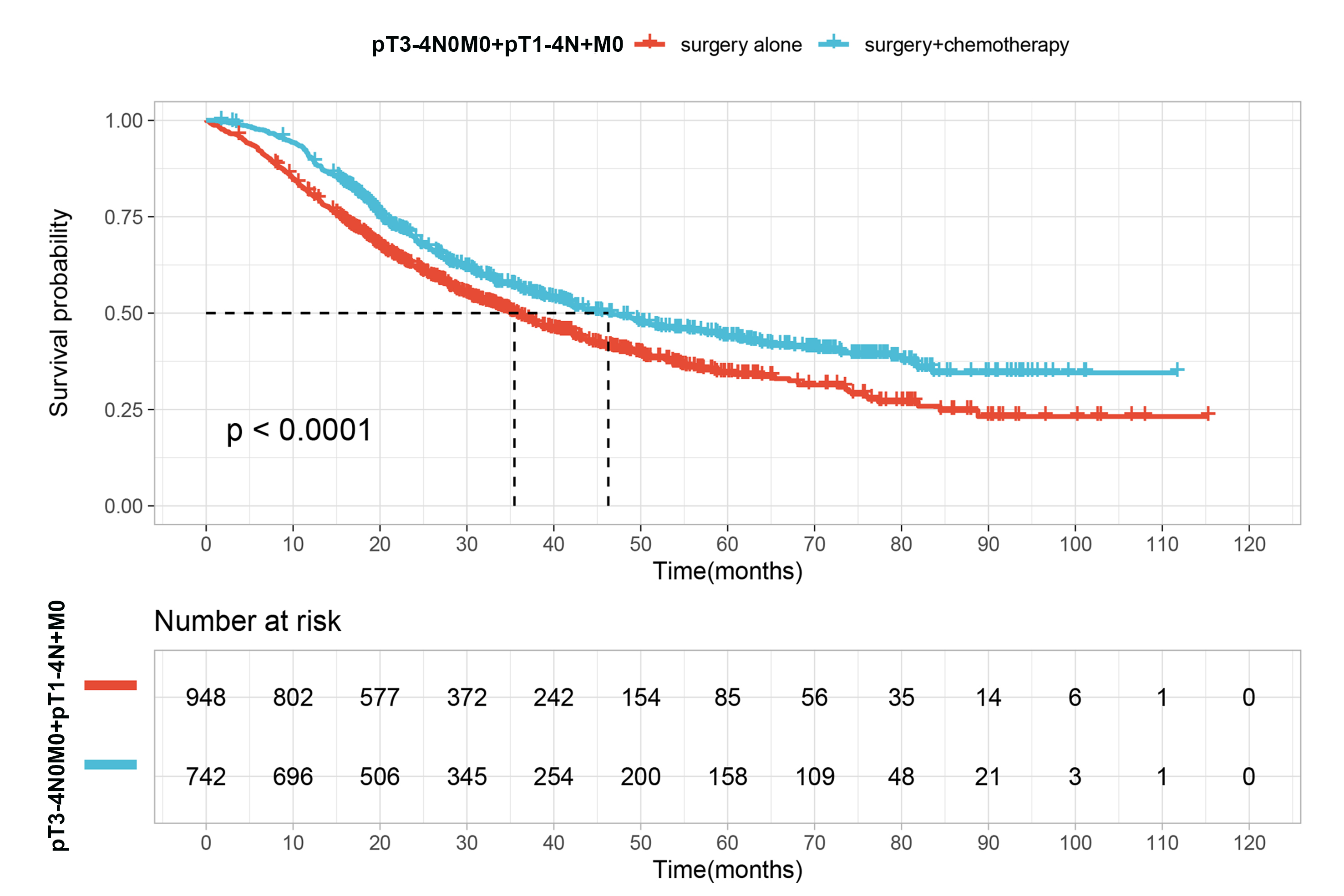

Supplement: Supplementary file 3 [file Image_3.jpeg]

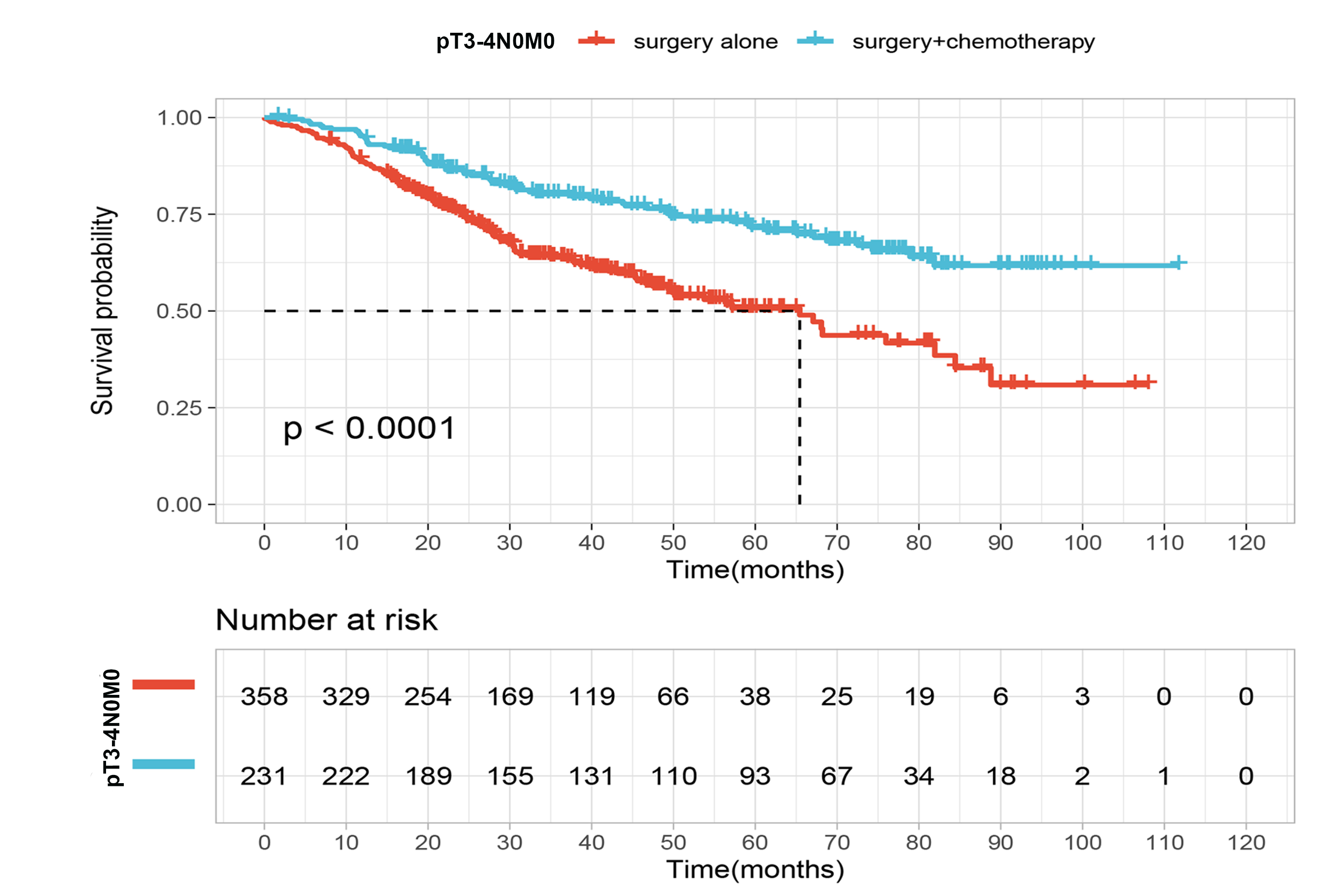

Supplement: Supplementary file 4 [file Image_4.jpeg]

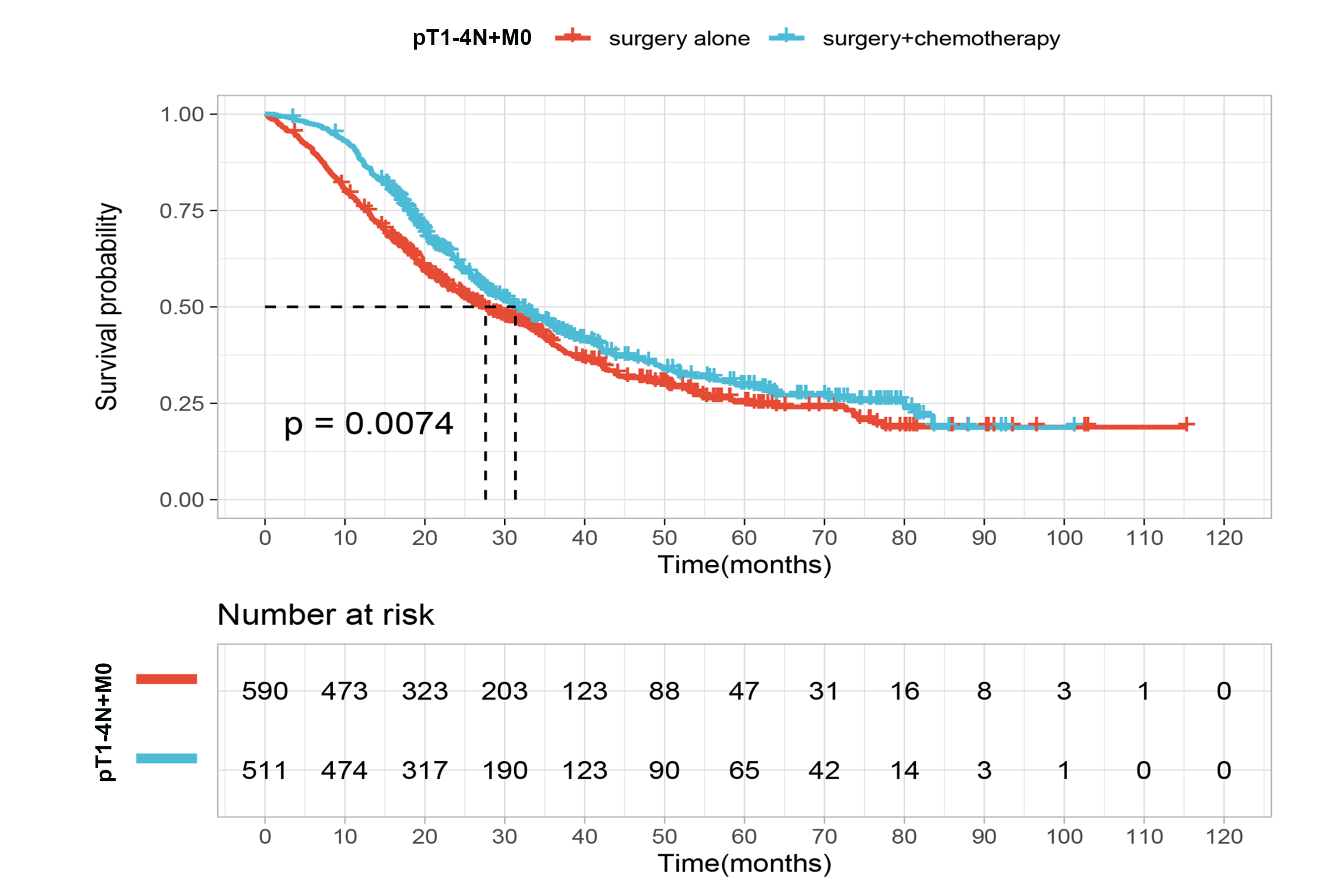

Supplement: Supplementary file 5 [file Image_5.jpeg]
